# Tetra­yttrium(III) tris­ulfide disilicate

**DOI:** 10.1107/S1600536810054607

**Published:** 2011-01-15

**Authors:** Lukasz A. Koscielski, James A. Ibers

**Affiliations:** a Department of Chemistry, Northwestern University, 2145 Sheridan Road, Evanston, IL 60208-3113, USA

## Abstract

Tetra­yttrium(III) tris­ulfide disilicate, Y_4_S_3_(Si_2_O_7_), crystallizes in the Sm_4_S_3_(Si_2_O_7_) structure type. The structure consists of isolated (Si_2_O_7_)^6−^ units (2*mm*. symmetry) and two crystallo­graphically independent Y^3+^ cations bridged by one S and one O atom. The first Y atom (site symmetry .*m*.) is coordinated by three O atoms and three S atoms in a trigonal–prismatic arrangement whereas the second Y atom (site symmetry ..2) is coordinated by six O atoms and three S atoms in a tricapped trigonal–prismatic arrangement.

## Related literature

For lanthanide sulfide disilicates of formula *Ln*_4_S_3_(Si_2_O_7_), see: Zeng *et al.* (1999 ▶) for *Ln* = La; Hartenbach & Schleid (2002 ▶) for *Ln* = Ce; Sieke & Schleid (2000 ▶) for *Ln* = Pr; Grupe *et al.* (1992 ▶) for *Ln* = Nd, Er; Sieke & Schleid (1999 ▶) for *Ln* = Sm; Sieke *et al.* (2002 ▶) for *Ln* = Gd, Tb, Dy, Ho, Er, Tm; Range *et al.* (1996 ▶) for *Ln* = Yb. For lanthanide selenide disilicates of formula *Ln*_4_Se_3_(Si_2_O_7_), see: Deudon *et al.* (1993 ▶) for *Ln* = La; Grupe & Urland (1989 ▶) for *Ln* = Ce, Nd; Grupe *et al.* (1992 ▶) for *Ln* = Pr, Sm, Gd. Ionic radii were taken from Shannon (1976 ▶). For computational details, see: Gelato & Parthé (1987 ▶). For additional synthetic details, see: Larroque & Beauvy (1986 ▶).

## Experimental

### 

#### Crystal data

Y_4_S_3_(Si_2_O_7_)
*M* *_r_* = 620.00
Tetragonal, 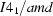

*a* = 11.6706 (16) Å
*c* = 13.5873 (19) Å
*V* = 1850.6 (4) Å^3^
*Z* = 8
Mo *K*α radiation
μ = 25.78 mm^−1^
*T* = 100 K
0.10 × 0.08 × 0.08 mm

#### Data collection

Bruker APEXII CCD diffractometer
Absorption correction: numerical [face-indexed using *SADABS* (Sheldrick, 2008*a* ▶)] *T*_min_ = 0.191, *T*_max_ = 0.238
10831 measured reflections
668 independent reflections
587 reflections with *I* > 2σ(*I*)
*R*_int_ = 0.066

#### Refinement

*R*[*F* ^2^ > 2σ(*F* ^2^)] = 0.020
*wR*(*F* ^2^) = 0.045
*S* = 1.25
668 reflections
47 parameters
Δρ_max_ = 0.61 e Å^−3^
Δρ_min_ = −0.77 e Å^−3^

### 

Data collection: *APEX2* (Bruker, 2009 ▶); cell refinement: *SAINT* (Bruker, 2009 ▶); data reduction: *SAINT*; program(s) used to solve structure: *SHELXS97* (Sheldrick, 2008*b* ▶); program(s) used to refine structure: *SHELXL97* (Sheldrick, 2008*b* ▶); molecular graphics: *CrystalMaker* (Palmer, 2009 ▶); software used to prepare material for publication: *SHELXL97*.

## Supplementary Material

Crystal structure: contains datablocks I, global. DOI: 10.1107/S1600536810054607/wm2440sup1.cif

Structure factors: contains datablocks I. DOI: 10.1107/S1600536810054607/wm2440Isup2.hkl

Additional supplementary materials:  crystallographic information; 3D view; checkCIF report

## Figures and Tables

**Table:** 

Y1—O2	2.279 (3)
Y1—O1^i^	2.428 (2)
Y1—S1^ii^	2.7714 (8)
Y1—S2	2.7874 (6)
Y2—O1^iii^	2.355 (2)
Y2—O2^iv^	2.3884 (15)
Y2—O1^iv^	2.530 (2)
Y2—S1^iv^	2.8419 (9)
Y2—S3^v^	2.8652 (6)
Si1—O3	1.621 (2)
Si1—O1	1.623 (2)
Si1—O2	1.641 (3)

**Table:** 

Y1^vi^—S1—Y2^vii^	90.389 (13)
Y1^viii^—O1—Y2^ix^	106.88 (8)
Si1^x^—O3—Si1	128.1 (3)

Symmetry codes: (i) 
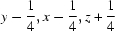
; (ii) 
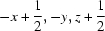
; (iii) 
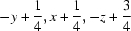
; (iv) 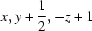; (v) 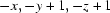; (vi) 
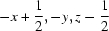
; (vii) 
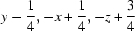
; (viii) 
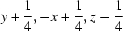
; (ix) 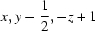; (x) 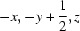.

**Fig. 1.**
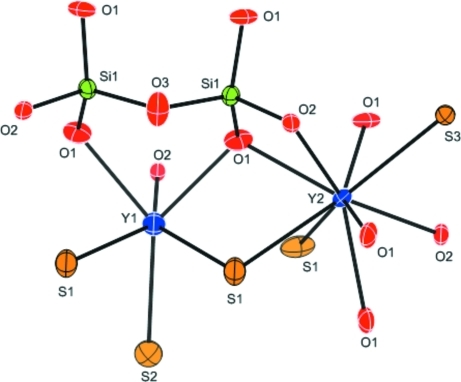
View showing the local coordination environment of atoms Y1 and Y2 as well as the disilicate unit. The 95% probability displacement ellipsoids are depicted.

**Fig. 2.**
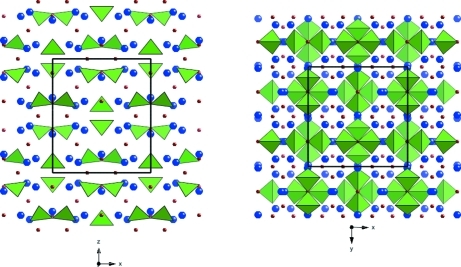
View down the b-axis (left) and down the c-axis (right). The disilicate units are staggered when viewed down the c-axis. Colour key: yttrium – blue, sulfur – brown, silicate tetrahedra – green. Unit cell is outlined.

**Fig. 3.**
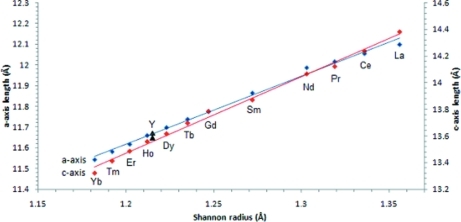
Plot of axial length versus lanthanide crystal radius for a 9-coordinate lanthanide in the Ln4S3(Si2O7) structure family (Ln = lanthanide element). Axial length decreases as the atomic mass of the lanthanide increases owing to the lanthanide contraction. Yttrium fits on the plot closest to holmium.

## supplementary crystallographic information

### Comment

Tetrayttrium(III) trisulfide disilicate, Y_4_S_3_(Si_2_O_7_), crystallizes in the Sm_4_S_3_(Si_2_O_7_) structure type (Grupe *et al.*, 1992). A view of the coordination environment of the atoms in Y_4_S_3_(Si_2_O_7_) is shown in Fig. 1. There are two crystallographically independent yttrium atoms. Atoms Y1 and Y2 are at sites of symmetry *.m.* and ..2, respectively. Atom Y1 is coordinated by three O atoms and three S atoms in a distorted trigonal-prismatic arrangement whereas atom Y2 is coordinated by six O atoms and three S atoms in the form of a distorted tri-capped trigonal prism. There are three crystallographically independent S atoms. Atoms S1, S2, and S3 are at sites of symmetry .2., 4*m*2, and 4*m*2, respectively. Atoms S1 and S2 are coordinated by four Y atoms in disphenoidal arrangements and atom S3 is coordinated by four Y atoms in a square-planar arrangement. There is one crystallographically independent Si atom at a site of symmetry .*m*. and three crystallographically independent O atoms at sites of symmetry 1, .*m*., and 2*mm*. . The disilicate (Si_2_O_7_)^6-^ units (symmetry 2*mm*.) are made up of two corner-sharing silicate tetrahedra in the form of a bow-tie. These units stack in a staggered fashion along the *c*-axis as seen in Fig. 2.

There exist eleven *Ln*_4_*Q*_3_(Si_2_O_7_) analogues where *Ln* is a lanthanide and *Q* is S, specifically when *Ln* = La–Nd, Sm, Gd–Tm (Zeng *et al.*, 1999; Hartenbach & Schleid, 2002; Sieke & Schleid, 1999; Grupe *et al.*, 1992; Sieke & Schleid, 1998; Sieke *et al.*, 2002; Range *et al.*, 1996). There exist six *Ln*_4_*Q*_3_(Si_2_O_7_) analogues of the title compound where *Q* = Se, specifically when *Ln* = La—Nd, Sm, Gd (Deudon *et al.*, 1993; Grupe & Urland, 1989; Grupe *et al.*, 1992). No analogues where *Q* = Te were found in the literature.

The title compound crystallizes with eight formula units in space group *I*4_1_/*amd*. The unit-cell dimensions are *a* = 11.6706 (16) Å and *c* = 13.5873 (19) Å. For the *Ln*_4_S_3_(Si_2_O_7_) analogues, the unit cell varies between *a* = 12.098 (3) Å and *c* = 14.379 (5) Å for *Ln* = La (Zeng *et al.*, 1999) and *a* = 11.543 (1) Å and *c* = 13.322 (1) Å for *Ln* = Yb (Range *et al.*, 1996). A plot of axis length *versus* lanthanide crystal radius (Shannon, 1976) leads to nearly linear curves (Sieke *et al.*, 2002) and adding *Ln* = Y to the plot not surprisingly keeps the near linearity. The plot is shown in Fig. 3. The unit-cell dimensions of Y_4_S_3_(Si_2_O_7_) are closest to that of Ho_4_S_3_(Si_2_O_7_), where *a* = 11.6595 (10) Å and *c* = 13.5577 (12) Å (Sieke *et al.*, 2002). In fact, of all the lanthanide radii, the crystal radius of Ho (1.212 Å) is closest to that of Y (1.215 Å) (Shannon, 1976).

### Experimental

The compound was synthesized accidentally. ThO_2_ (Alfa-Aesar), Y_2_S_3_ (Strem, 99.9%) S (Alfa-Aesar, 99.99%), and Sb (Aldrich, 99.5%), were used as received. Sb_2_S_3_ was prepared from the direct reaction of the elements in a sealed fused-silica tube at 1123 K. ThOS was prepared from ThO_2_ and S following a modified procedure by Larroque *et al.* (1986). A fused-silica tube was loaded with ThOS (35 mg, 0.125 mmol) and Y_2_S_3_ (35.6 mg, 0.130 mmol), evacuated to near 10 ^-4^ Torr, flame sealed, and placed in a computer-controlled furnace. It was heated to 1273 K in 24 h, kept at 1273 K for 168 h, cooled to 873 K in 198 h, and then rapidly cooled to 298 K in 5 h. The resulting tan powder (50 mg) was loaded with Sb_2_S_3_ (20 mg, 0.6 mmol) in a fused-silica tube and heated as before. The resulting tube was etched and contained clear crystals of composition Y/S/Si/O as determined by EDX analysis. The silicon and oxygen were abstracted from the silica tube and introduced into the reaction in the second step.

### Refinement

Origin choice 2 of space group *I*4_1_/*amd* was used. The structure was standardized by means of the program *STRUCTURE TIDY* (Gelato & Parthé, 1987). The highest peak (0.61 (16) e Å^-3^) is 0.48 Å from atom O3 and the deepest hole (-0.77 (16) e Å^-3^) is 0.45 Å from atom Y1.

### Crystal data

**Table:**

Y_4_S_3_(Si_2_O_7_)	*D*_x_ = 4.451 Mg m^−^^3^
*M**_r_* = 620.00	Mo *K*α radiation, λ = 0.71073 Å
Tetragonal, *I*4_1_/*a**m**d*	Cell parameters from 2730 reflections
Hall symbol: -I 4bd 2	θ = 2.3–27.6°
*a* = 11.6706 (16) Å	µ = 25.78 mm^−^^1^
*c* = 13.5873 (19) Å	*T* = 100 K
*V* = 1850.6 (4) Å^3^	Polyhedron, colorless
*Z* = 8	0.10 × 0.08 × 0.08 mm
*F*(000) = 2304	

### Data collection

**Table:**

Bruker APEXII CCD diffractometer	668 independent reflections
Radiation source: fine-focus sealed tube	587 reflections with *I* > 2σ(*I*)
graphite	*R*_int_ = 0.066
ω scans	θ_max_ = 29.2°, θ_min_ = 2.3°
Absorption correction: numerical [face-indexed using *SADABS* (Sheldrick, 2008*a*)]	*h* = −15→15
*T*_min_ = 0.191, *T*_max_ = 0.238	*k* = −15→15
10831 measured reflections	*l* = −18→18

### Refinement

**Table:**

Refinement on *F*^2^	Primary atom site location: structure-invariant direct methods
Least-squares matrix: full	Secondary atom site location: difference Fourier map
*R*[*F*^2^ > 2σ(*F*^2^)] = 0.020	*w* = [1/[σ^2^(*F*_o_^2^) + (0.0199**F*_o_^2^)^2^]
*wR*(*F*^2^) = 0.045	(Δ/σ)_max_ = 0.001
*S* = 1.25	Δρ_max_ = 0.61 e Å^−^^3^
668 reflections	Δρ_min_ = −0.77 e Å^−^^3^
47 parameters	Extinction correction: *SHELXL97* (Sheldrick, 2008*a*), Fc^*^=kFc[1+0.001xFc^2^λ^3^/sin(2θ)]^-1/4^
0 restraints	Extinction coefficient: 0.00065 (7)

### Fractional atomic coordinates and isotropic or equivalent isotropic displacement parameters (Å^2^)

**Table:**

	*x*	*y*	*z*	*U*_iso_*/*U*_eq_	
Y1	0.0000	0.01464 (4)	0.34012 (3)	0.00768 (13)	
Y2	0.17360 (2)	0.42360 (2)	0.8750	0.00517 (13)	
S1	0.35327 (9)	0.0000	0.0000	0.0096 (2)	
S2	0.0000	0.2500	0.3750	0.0089 (4)	
S3	0.0000	0.7500	0.1250	0.0052 (4)	
Si1	0.0000	0.12512 (10)	0.09531 (9)	0.0049 (2)	
O1	0.12244 (17)	0.10968 (19)	0.04018 (15)	0.0082 (5)	
O2	0.0000	0.0169 (2)	0.1724 (2)	0.0062 (6)	
O3	0.0000	0.2500	0.1475 (3)	0.0110 (10)	

### Atomic displacement parameters (Å^2^)

**Table:**

	*U*^11^	*U*^22^	*U*^33^	*U*^12^	*U*^13^	*U*^23^
Y1	0.0078 (2)	0.0087 (2)	0.0065 (2)	0.000	0.000	−0.00089 (16)
Y2	0.00547 (15)	0.00547 (15)	0.0046 (2)	0.00152 (15)	0.00013 (11)	−0.00013 (11)
S1	0.0059 (5)	0.0152 (6)	0.0077 (6)	0.000	0.000	0.0041 (4)
S2	0.0098 (7)	0.0098 (7)	0.0072 (11)	0.000	0.000	0.000
S3	0.0048 (6)	0.0048 (6)	0.0062 (10)	0.000	0.000	0.000
Si1	0.0055 (6)	0.0041 (5)	0.0050 (6)	0.000	0.000	0.0006 (4)
O1	0.0044 (10)	0.0116 (11)	0.0085 (12)	−0.0005 (9)	0.0019 (8)	0.0017 (9)
O2	0.0036 (14)	0.0048 (14)	0.0103 (18)	0.000	0.000	−0.0008 (12)
O3	0.020 (2)	0.007 (2)	0.006 (2)	0.000	0.000	0.000

### Geometric parameters (Å, °)

**Table:**

Y1—O2	2.279 (3)	S1—Y1^xvii^	2.7714 (8)
Y1—O1^i^	2.428 (2)	S1—Y2^xviii^	2.8420 (9)
Y1—O1^ii^	2.428 (2)	S1—Y2^vii^	2.8420 (9)
Y1—S1^iii^	2.7714 (8)	S2—Y1^x^	2.7874 (6)
Y1—S1^iv^	2.7714 (8)	S2—Y1^xviii^	2.7874 (6)
Y1—S2	2.7874 (6)	S2—Y1^xix^	2.7874 (6)
Y1—Si1^v^	3.4158 (8)	S3—Y2^xii^	2.8652 (5)
Y1—Si1^ii^	3.4158 (8)	S3—Y2^xv^	2.8652 (6)
Y1—Y2^vi^	3.7117 (5)	S3—Y2^xx^	2.8652 (6)
Y1—Y2^vii^	3.7117 (5)	S3—Y2^xxi^	2.8652 (5)
Y1—Y2^viii^	3.9830 (6)	Si1—O3	1.621 (2)
Y1—Y2^ix^	3.9830 (6)	Si1—O1	1.623 (2)
Y2—O1^x^	2.355 (2)	Si1—O1^xxii^	1.623 (2)
Y2—O1^xi^	2.355 (2)	Si1—O2	1.641 (3)
Y2—O2^xii^	2.3884 (15)	Si1—Y2^vi^	3.1303 (10)
Y2—O2^xiii^	2.3884 (15)	Si1—Y2^vii^	3.1303 (10)
Y2—O1^xii^	2.530 (2)	Si1—Y1^xxiii^	3.4158 (8)
Y2—O1^xiv^	2.530 (2)	Si1—Y1^xxiv^	3.4158 (8)
Y2—S1^xii^	2.8419 (9)	O1—Y2^xviii^	2.355 (2)
Y2—S1^x^	2.8419 (9)	O1—Y1^xxiii^	2.428 (2)
Y2—S3^xv^	2.8652 (6)	O1—Y2^vii^	2.530 (2)
Y2—Si1^xiii^	3.1303 (10)	O2—Y2^vi^	2.3884 (15)
Y2—Si1^xii^	3.1303 (10)	O2—Y2^vii^	2.3884 (15)
Y2—Y1^xii^	3.7117 (5)	O3—Si1^xix^	1.621 (2)
S1—Y1^xvi^	2.7714 (8)		
			
O2—Y1—O1^i^	74.24 (7)	O1^xi^—Y2—S3^xv^	72.79 (6)
O2—Y1—O1^ii^	74.24 (7)	O2^xii^—Y2—S3^xv^	73.89 (6)
O1^i^—Y1—O1^ii^	84.81 (11)	O2^xiii^—Y2—S3^xv^	73.89 (6)
O2—Y1—S1^iii^	141.68 (2)	O1^xii^—Y2—S3^xv^	116.11 (5)
O1^i^—Y1—S1^iii^	126.41 (5)	O1^xiv^—Y2—S3^xv^	116.11 (5)
O1^ii^—Y1—S1^iii^	76.13 (5)	S1^xii^—Y2—S3^xv^	138.038 (9)
O2—Y1—S1^iv^	141.68 (2)	S1^x^—Y2—S3^xv^	138.038 (9)
O1^i^—Y1—S1^iv^	76.13 (5)	Y1^xvi^—S1—Y1^xvii^	103.68 (4)
O1^ii^—Y1—S1^iv^	126.41 (5)	Y1^xvi^—S1—Y2^xviii^	154.823 (15)
S1^iii^—Y1—S1^iv^	76.32 (4)	Y1^xvii^—S1—Y2^xviii^	90.389 (13)
O2—Y1—S2	99.13 (7)	Y1^xvi^—S1—Y2^vii^	90.389 (13)
O1^i^—Y1—S2	136.14 (5)	Y1^xvii^—S1—Y2^vii^	154.823 (15)
O1^ii^—Y1—S2	136.14 (5)	Y2^xviii^—S1—Y2^vii^	84.90 (3)
S1^iii^—Y1—S2	85.841 (11)	Y1^x^—S2—Y1^xviii^	160.421 (18)
S1^iv^—Y1—S2	85.841 (11)	Y1^x^—S2—Y1^xix^	91.657 (3)
O1^x^—Y2—O1^xi^	145.57 (11)	Y1^xviii^—S2—Y1^xix^	91.657 (3)
O1^x^—Y2—O2^xii^	73.66 (8)	Y1^x^—S2—Y1	91.657 (3)
O1^xi^—Y2—O2^xii^	96.72 (8)	Y1^xviii^—S2—Y1	91.657 (3)
O1^x^—Y2—O2^xiii^	96.72 (8)	Y1^xix^—S2—Y1	160.420 (18)
O1^xi^—Y2—O2^xiii^	73.66 (8)	Y2^xii^—S3—Y2^xv^	180.0
O2^xii^—Y2—O2^xiii^	147.78 (12)	Y2^xii^—S3—Y2^xx^	90.0
O1^x^—Y2—O1^xii^	127.80 (7)	Y2^xv^—S3—Y2^xx^	90.0
O1^xi^—Y2—O1^xii^	69.36 (8)	Y2^xii^—S3—Y2^xxi^	90.0
O2^xii^—Y2—O1^xii^	62.05 (8)	Y2^xv^—S3—Y2^xxi^	90.0
O2^xiii^—Y2—O1^xii^	135.47 (8)	Y2^xx^—S3—Y2^xxi^	180.0
O1^x^—Y2—O1^xiv^	69.36 (8)	O3—Si1—O1	107.56 (10)
O1^xi^—Y2—O1^xiv^	127.80 (7)	O3—Si1—O1^xxii^	107.56 (10)
O2^xii^—Y2—O1^xiv^	135.47 (8)	O1—Si1—O1^xxii^	123.34 (16)
O2^xiii^—Y2—O1^xiv^	62.05 (8)	O3—Si1—O2	114.39 (18)
O1^xii^—Y2—O1^xiv^	127.78 (9)	O1—Si1—O2	102.07 (10)
O1^x^—Y2—S1^xii^	140.43 (6)	O1^xxii^—Si1—O2	102.07 (10)
O1^xi^—Y2—S1^xii^	70.68 (5)	Si1—O1—Y2^xviii^	132.95 (12)
O2^xii^—Y2—S1^xii^	130.09 (7)	Si1—O1—Y1^xxiii^	113.44 (11)
O2^xiii^—Y2—S1^xii^	76.63 (6)	Y2^xviii^—O1—Y1^xxiii^	101.79 (7)
O1^xii^—Y2—S1^xii^	68.44 (5)	Si1—O1—Y2^vii^	95.33 (10)
O1^xiv^—Y2—S1^xii^	73.32 (5)	Y2^xviii^—O1—Y2^vii^	103.46 (8)
O1^x^—Y2—S1^x^	70.68 (5)	Y1^xxiii^—O1—Y2^vii^	106.88 (8)
O1^xi^—Y2—S1^x^	140.43 (6)	Si1—O2—Y1	130.32 (16)
O2^xii^—Y2—S1^x^	76.63 (6)	Si1—O2—Y2^vi^	100.30 (9)
O2^xiii^—Y2—S1^x^	130.09 (7)	Y1—O2—Y2^vi^	105.32 (8)
O1^xii^—Y2—S1^x^	73.32 (5)	Si1—O2—Y2^vii^	100.30 (9)
O1^xiv^—Y2—S1^x^	68.44 (5)	Y1—O2—Y2^vii^	105.32 (8)
S1^xii^—Y2—S1^x^	83.925 (17)	Y2^vi^—O2—Y2^vii^	116.05 (12)
O1^x^—Y2—S3^xv^	72.79 (6)	Si1^xix^—O3—Si1	128.1 (3)

Symmetry codes: (i) *y*−1/4, *x*−1/4, *z*+1/4; (ii) −*y*+1/4, *x*−1/4, *z*+1/4; (iii) −*x*+1/2, −*y*, *z*+1/2; (iv) *x*−1/2, *y*, −*z*+1/2; (v) *y*−1/4, −*x*−1/4, *z*+1/4; (vi) −*y*+1/4, *x*−1/4, *z*−3/4; (vii) *x*, *y*−1/2, −*z*+1; (viii) *x*−1/2, *y*−1/2, *z*−1/2; (ix) −*y*+3/4, *x*−1/4, −*z*+5/4; (x) −*y*+1/4, *x*+1/4, −*z*+3/4; (xi) *x*, −*y*+1/2, *z*+1; (xii) *x*, *y*+1/2, −*z*+1; (xiii) *y*+1/4, −*x*+1/4, *z*+3/4; (xiv) *y*+1/4, *x*+1/4, *z*+3/4; (xv) −*x*, −*y*+1, −*z*+1; (xvi) *x*+1/2, *y*, −*z*+1/2; (xvii) −*x*+1/2, −*y*, *z*−1/2; (xviii) *y*−1/4, −*x*+1/4, −*z*+3/4; (xix) −*x*, −*y*+1/2, *z*; (xx) *y*−1/4, −*x*+3/4, *z*−3/4; (xxi) −*y*+1/4, *x*+3/4, *z*−3/4; (xxii) −*x*, *y*, *z*; (xxiii) *y*+1/4, −*x*+1/4, *z*−1/4; (xxiv) −*y*−1/4, *x*+1/4, *z*−1/4.
